# Replacement of connexin43 by connexin26 in transgenic mice leads to dysfunctional reproductive organs and slowed ventricular conduction in the heart

**DOI:** 10.1186/1471-213X-7-26

**Published:** 2007-04-04

**Authors:** Elke Winterhager, Nicole Pielensticker, Jennifer Freyer, Alexander Ghanem, Jan W Schrickel, Jung-Sun Kim, Rüdiger Behr, Ruth Grümmer, Karen Maass, Stephanie Urschel, Thorsten Lewalter, Klaus Tiemann, Manuela Simoni, Klaus Willecke

**Affiliations:** 1 Institut für Anatomie, Universität Duisburg-Essen, 45122 Essen, Germany; 2 Institut für Genetik, Universität Bonn, 53117 Bonn, Germany; 3 Medizinische Klinik und Poliklinik II, Universitätsklinikum Bonn, 53105 Bonn, Germany; 4 University of Ulsan, College of Medicine, Seoul, 138-736 Republic of Korea; 5 Institut für Reproduktive Medizin, Universität Münster, 48149 Münster, Germany

## Abstract

**Background:**

In order to further distinguish unique from general functions of connexin43, we have generated mice in which the coding region of connexin43 was replaced by that of connexin26.

**Results:**

Heterozygous mothers showed impaired mammary gland development responsible for decreased lactation and early postnatal death of the pups which could be partially rescued by wild type foster mothers. Only about 17% of the homozygous connexin43 knock-in connexin26 mice instead of 25% expected according to Mendelian inheritance, were born and only 6% survived to day 21 post partum and longer. Neonatal and adult connexin43 knock-in connexin26 mice exhibited slowed ventricular conduction in their hearts, i.e. similar but delayed electrophysiological abnormalities as connexin43 deficient mice. Furthermore, connexin43 knock-in connexin26 male and female mice were infertile and exhibited hypotrophic gonads. In testes, tubuli seminiferi were developed and spermatogonia as well as some primary spermatocytes were present, but further differentiated stages of spermatogenesis were absent. Ovaries of female connexin43 knock-in connexin26 mice revealed only few follicles and the maturation of follicles was completely impaired.

**Conclusion:**

The impaired gametogenesis of homozygous males and females can explain their infertility.

## Background

Connexins form intercellular conduits permitting diffusional exchange of ions, secondary messenger molecules and metabolites up to 1000 Dalton. These exchange properties regulate and coordinate several cell biological functions such as cell growth, differentiation and developmental processes [1]. Six connexin protein subunits form a hemichannel, also called connexon. Two docking connexons, contributed by two adjacent cells, form a gap junctional channel. To date, 20 different connexin genes have been identified in the mouse genome [2]. Connexins can assemble into homomeric or heteromeric hemichannels, which in turn can dock to hemichannels of the same or different connexin composition in the plasma membrane of contacting cells to form homotypic or heterotypic channels [3]. Gap junction channels composed of different connexin isoforms differ from each other in unitary conductance and permeability to second messenger molecules [4]. This raised the question to which extent the different connexin isoforms limit or support functional specialization of different cell types.

Of the known connexin (Cx) genes, Cx43 is most abundantly expressed in many cell types. In mouse heart, Cx43 protein is found in the working myocardium and in Purkinje fibers [5]. Cx43 deficient mice die shortly after birth due to obstruction of right ventricular outflow tract of the heart [6]. In reproductive organs Cx43 has been shown to control proliferation of granulosa cells [7]. In the testis, Cx43 containing gap junction channels connect the Leydig as well as the Sertoli cells and loss of Cx43 seems to impair spermatogenesis [8]. It was described that postnatal lethality of Cx43 deficient mice could be rescued by Cx32 or Cx40, indicating that Cx43, Cx40 and Cx32 share some common functions [9]. Nevertheless, Cx43 knock-in Cx32 (Cx43KI32) and Cx43 knock-in Cx40 (Cx43KI40) mice showed functional and morphological differences, compared to wild-type mice.

Cx26 is one of the two major gap junction proteins expressed in hepatocytes. It was also found in placenta [10], in the epidermis, and in several other organs, including support cells of the inner ear [11], and in the uterine epithelium during pregnancy in response to embryo recognition [12]. In mammary tissue, Cx26 and Cx32 are present in the secretory epithelium and Cx43 is localized in the myoepithelium of the mammary gland [13,14] as well as in several cell lines suggesting that these connexins might be important for development during pregnancy and function during lactation [15]. Cx26 and Cx32 have been co-localized in gap junctions of mouse mammary epithelium during lactation and both homomeric and heteromeric connexins have been identified [16]. The unique expression patterns of Cx26 and Cx32 suggest distinct and overlapping roles in mammary development and function. Cx26 mRNA and protein were first detected in mouse mammary tissue on day 4 of pregnancy which was followed by a steady increase and maximal expression during lactation [13,16]. In contrast, a significant induction of Cx32 expression occurred within a few hours after parturition [16].

The analysis of connexin knock-in mice is a useful strategy to identify unique features of different connexins and to distinguish them from properties shared by several connexin channels. Especially the Cx43 locus was used several times for the generation of knock-in mice. The phenotypes of Cx43KI32, Cx43KI40 and Cx43KI31 mice have been investigated [9,17]. The regulatory consequences of Cx43 phosphorylation were investigated in several laboratories [18]. Here we have replaced the coding region of Cx43 by that of Cx26. The non-phosphorylated Cx26 protein is of special interest, since it is possible to gain insights into the function of connexin channels independent of the regulation by phosphorylation.

Furthermore, we wanted to clarify whether the replacement of Cx43 by Cx26 causes a similar phenotype as observed with homozygous Cx43KI32 mice, i.e. severe impairment of spermatogenesis. In addition, heterozygous Cx43KI32 females exhibited problems with lactation. It had been hypothesized that this phenotype could be due to defects in cooperation between myoepithelial and glandular cells [9].

## Results

### Generation and transgenic expression analysis of Cx43KI26neo mice

**Figure 1:**
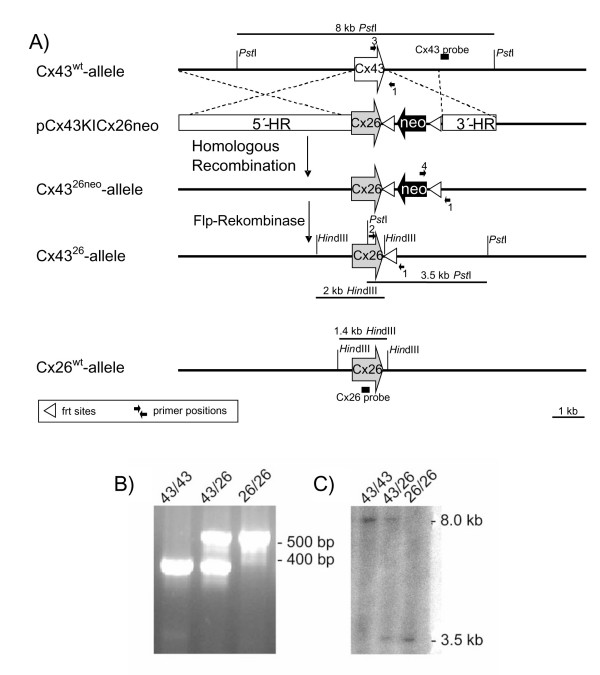
Generation and characterization of Cx43KI26 mice. (A) pCx43KI26 vector DNA was transfected into HM-1 embryonic stem cells. Homologous recombination was tested by PCR and Southern blot analyses. Homozygously recombined clones were injected into blastocysts of C57BL/6 mice, in order to generate first chimeras and in the next generation of mice carrying the Cx43KI26neo allele. By means of Flp activity, the frt-flanked selection cassette was deleted, resulting in the Cx43KI26 allele. In mice carrying this allele, Cx26 is expressed under control of Cx43 regulatory elements. (B) Multiplex PCR of different genotypes using primer 1 (Cx43-RO4), primer 2 (Ki26Cx26) and primer 3 (Cx43-HO2). A 381 bp amplicon indicates the wild type Cx43 allele and a 529 bp fragment the Ca43KI26 allele. The middle lane represents the heterozygous genotype Cx43^43/26^. (C) Southern blot analysis of *Pst*I digested Cx43^43/43^, Cx43^43/26 ^and Cx43^26/26 ^DNA using the external Cx43 probe. A 8 kb fragment is indicative of the Cx43 wild-type allele, whereas a 3.5 kb fragment represents the Cx43KI26 allele.

In order to generate Cx43^26/26 ^mice, we replaced the Cx43 coding DNA by Cx26 coding DNA followed by a neomycin resistance cassette in HM-1 embryonic stem (ES) cells. The coding region of Cx26 was cloned behind the translational start codon of Cx43. For this purpose an additional NcoI restriction site was created by insertion of an additional cytosine residue using modified primers (Figure 1A). Three correctly recombined ES cell clones gave rise to chimeras that transmitted the mutant allele through the germ line when injected into blastocysts. Heterozygous, male Cx43^43/26 ^mice were crossed with female Flp recombinase expressing mice. The Flp-mediated recombination led to excision of the PGK-neomycin cassette. The heterozygous mice gave birth to homozygous mutant offspring (i.e. Cx43^26/26^) when intercrossed, as demonstrated by Southern blot and PCR analyses (Figure 1B–C). A multiplex PCR was established for genotyping using a common reverse primer directed to the 3' UTR of Cx43. A forward primer directed to the coding region of Cx43 and another one to the coding region of Cx26 were employed. A 381 amplicon was derived from the wild-type allele and a 529 bp amplicon was derived from the Cx43 knock-in Cx26 allele (Figure 1B). In the Southern blot hybridization, the Cx43 external probe recognized a 8 kb wild-type fragment and a 3.5 kb knock-in fragment (Figure 1C).

**Figure 2:**
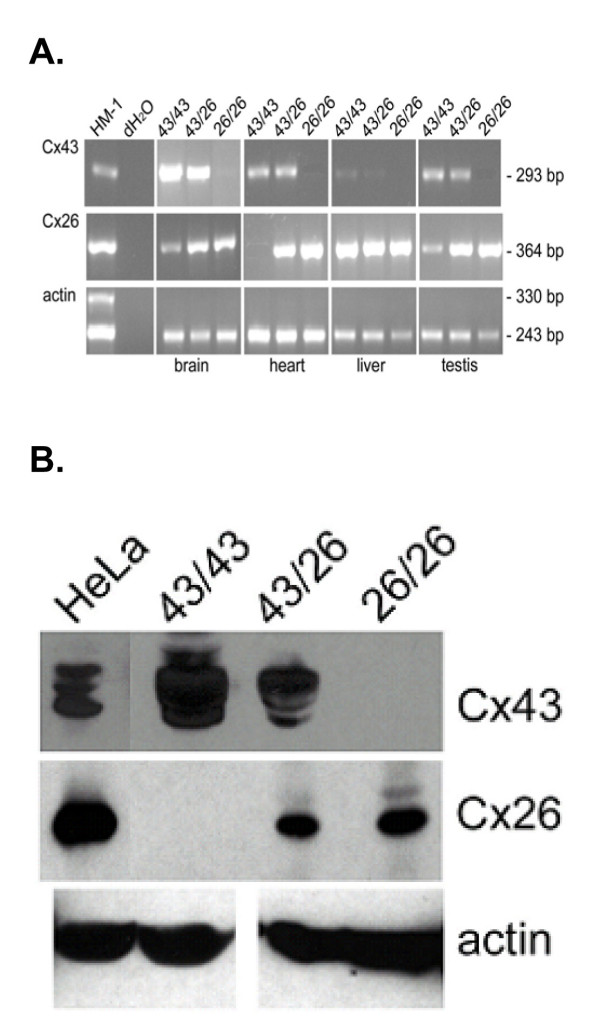
A. RT-PCR analyses of Cx43 and Cx26 mRNA expression in brain, heart, liver and testis. Fragments: Cx43: 293 bp, Cx26: 364 bp. RNA from HM-1 embryonic stem cells and distilled water served as controls. Equal loading was demonstrated by actin amplicons (243 bp). B. Western blot analyses of Cx43 and Cx26 protein expression in heart lysates. Cx43 protein was not detected in homozygous Cx43KI26 animals (lane 3). Cx26 was not found in wild-type heart (43/43), whereas it was expressed in heterozygous (43/26) mice and at nearly the double amount in homozygous Cx43KI26 animals. Loading of equal amounts of homogenates was verified by re-immunolabeling of the same membrane with monoclonal anti-tubulin.

Intron spanning RT-PCR analyses were used to demonstrate spliced isoforms, transcribed from the different Cx43 alleles. The mRNA from brain, heart, liver and testis was transcribed in cDNA, and the PCRs were carried out with different primers for Cx43, Cx26 and β-actin (Figure 2A). As expected, the primers yielded an amplicon of 293 bp for Cx43, an amplicon of 364 bp for Cx26 and an amplicon of 243 bp for β-actin (control). The Cx43 amplicon was found in brain, heart and testis of wild-type and heterozygous mice (Cx43^43/26^), but only weakly in liver where Ito cells and cholangiocytes but not hepatocytes express Cx43. Cx26 was expressed in brain, liver as well as testis of all mice due to endogenous expression and in particular in hearts of heterozygous Cx43^43/26 ^as well as homozygous Cx43^26/26 ^mice. As expected, wild-type mice Cx43^43/43 ^did not express Cx26 mRNA in their hearts.

Expression of the Cx26 protein was confirmed by Western blot analysis (Figure 2B) in the hearts of heterozygous Cx43^43/26 ^and homozygous Cx43^26/26 ^mice. No Cx26 protein was detected in hearts of homozygous Cx43^43/43 ^mice.

### Dominant impairment of mammary function in Cx43^43/26 ^heterozygous mice

**Figure 3:**
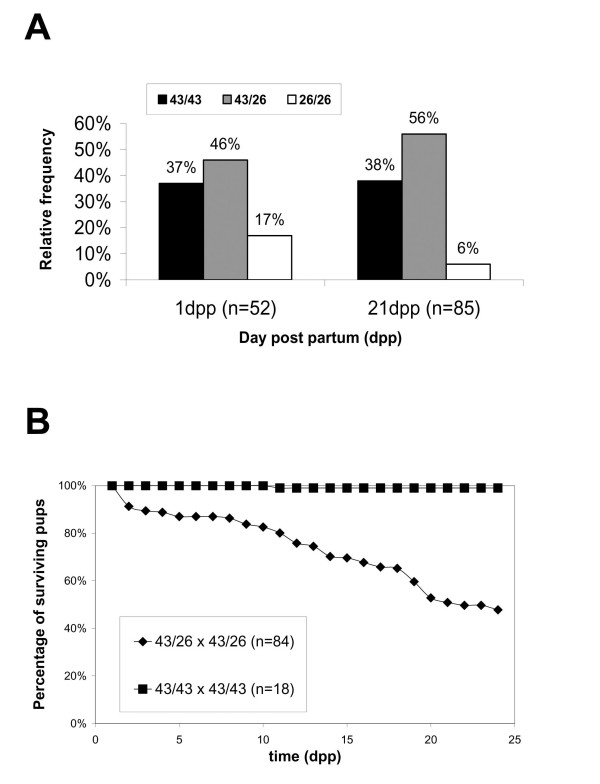
Postnatal survival frequency of pups in litters after crossing of heterozygous Cx43^43/26 ^mice. A. Mendelian frequencies of wild type (Cx43^43/43^), heterozygous (Cx43^43/26^) and homozygous (Cx43^26/26^) mice at 1 dpp and 21 dpp; n, number of mice analyzed. B, Postnatal survival of litters (all genotypes summarized) after mating of heterozygous Cx43^43/26 ^mice or wild type Cx43^43/43 ^mice; n, number of litters analyzed.

Only about 17% of the homozygous Cx43^26/26 ^mice instead of 25%, expected according to Mendelian inheritance, were born and only 6% (instead of 25%) survived to day 21 post partum and longer (Figure 3A). Preferential loss of homozygous Cx43^26/26 ^animals coincided with general high mortality in the offspring of heterozygous Cx43^43/26 ^parents (Figure 3B). This high mortality correlated with the maternal heterozygous Cx43^43/26 ^phenotype. The surviving heterozygous adult mice (>10 weeks) were indistinguishable in their appearance from wild type litter mates, whereas homozygous males and females weighed about 30% less.

The generally high mortality among the pups of heterozygous Cx43^43/26 ^mothers was accompanied by decreased postnatal growth, and continuous loss of the pups, suggesting that the pups died from starvation This phenomenon was already observed with heterozygous Cx43^43/32 ^mothers, but was not reported for heterozygous Cx43-deficient (Cx43^+/-^) mice [6], confirming the dominant and specific mode of inheritance of the Cx43^43/26 ^allele. Maternal behaviour of heterozygous Cx43^43/26 ^mothers did not differ from controls. When newborn litters of Cx43^43/26 ^mothers were raised by wild type Cx43^43/43 ^foster mothers, survival of the pups was highly improved. This procedure had to be carried out, in order to expand the Cx43^43/26 ^mouse colony but it was not quantitated due to occasional loss of pups not accepted by the foster mothers.

**Figure 4:**
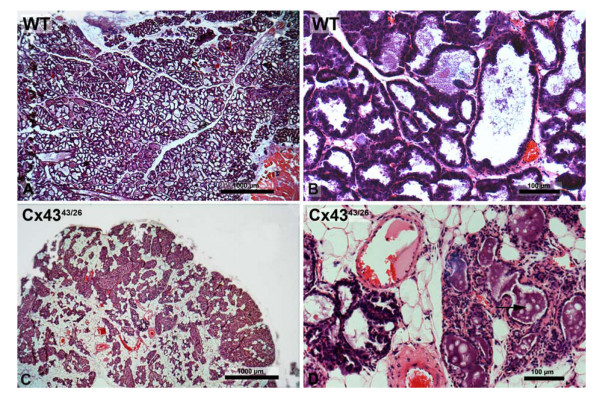
Histological section of the lactating mammary gland of wild-type (A, B) and heterozygous Cx26 knock-in (C, D). The. In contrast to the wild-type mammary glands, heterozygous Cx43^43/26 ^glands show a dramatically reduced branching of the ductuli. As a consequence, less alveoli surrounded by adipocytic tissue were seen (compare A with C). The alveoli were mostly smaller in diameter but did show apocrine secretion and milk droplets in the lumen (arrow) (D).

These observations suggested an insufficient nutrition due to an impaired mammary gland function during lactation. In comparison to wild-type mice, histological analysis of heterozygous Cx43^43/26 ^mothers (9 dpc, 8 pups) revealed that the glandular alveoli were less dilated and the ductuli were obviously less branched resulting in lower amount of secretory alveoli. Instead, extended areas of adipose tissue were observed between the glandular units (compare Figure 4A with 4C). Higher magnification confirmed secretory transformation of alveolar epithelium with dilated alveoli and the typical apocrine protrusions as well as milk droplets in the alveoli (Figure 4B–D). Both the presence of milk droplets found in alveoli and the histological features suggested that reduced development of the glands rather than milk production was impaired in heterozygous Cx43^43/26 ^mothers.

### Infertility of homozygous Cx43^26/26 ^mice

**Figure 5:**
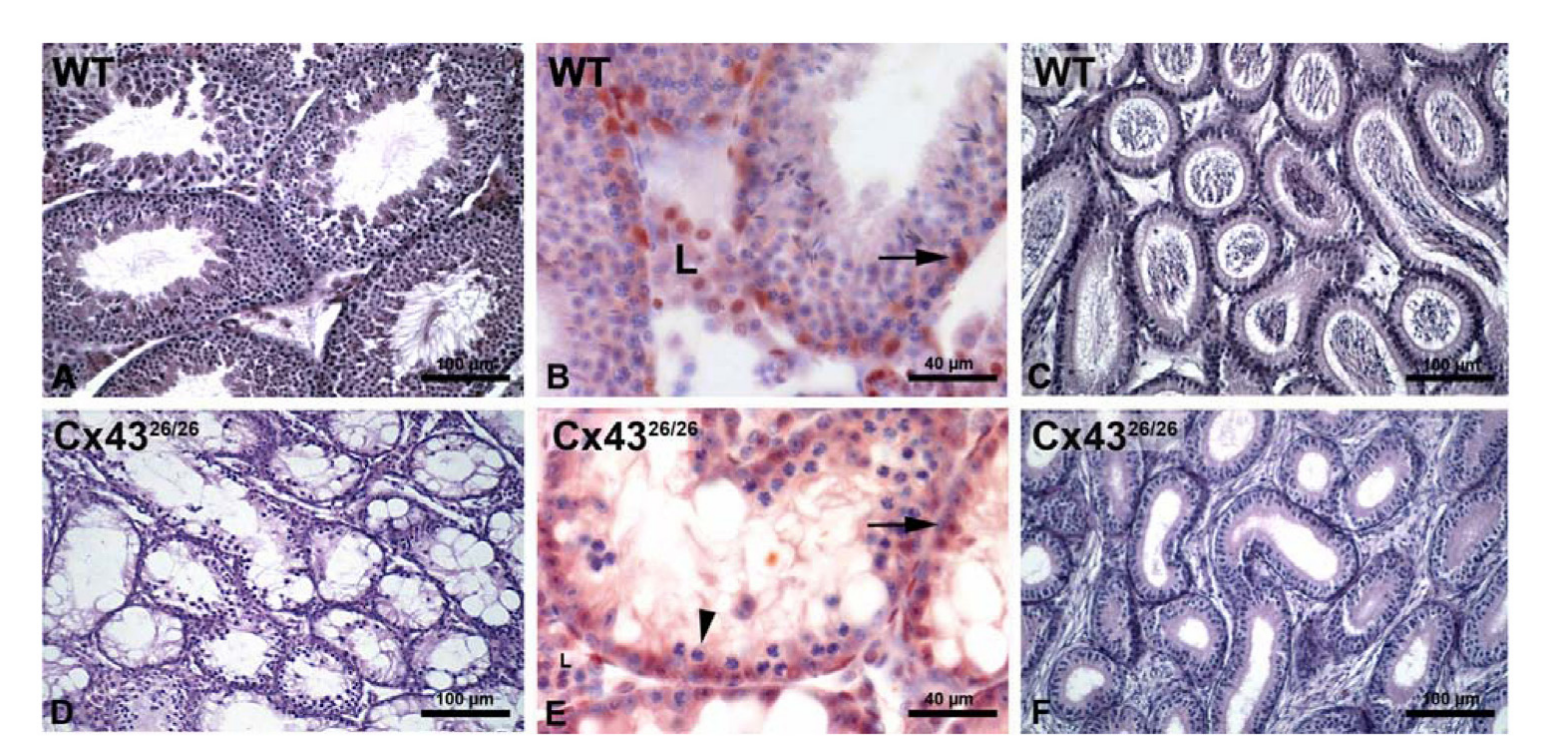
Tubuli seminiferi (A, B) and ductuli epididymidis (C) of wild-type and Cx43^26/26 ^mice (D-F). Testes of wild-type males revealed densely packed tubuli seminiferi with intact spermatogenensis and ductuli epididymis filled with mature sperms. In Cx43^26/26 ^mice, the seminiferous tubules demonstrated absence of mature sperms but a germinal epithelium. The loss of spermiogenesis was indicated by an empty epididymis with less densely packed tubules. Like in controls (B) immunolabeling of the testosterone receptor revealed the presence of Sertoli (arrows) and Leydig cells in testes of Cx43^26/26 ^mice (E). Primary spermatogonia and spermatocytes type 1 (arrowhead) were present but there was no complete spermatogenesis (E). L, Leydig cells.

Histological analysis of lung, thymus, pancreas, spleen, gastrointestinal tract, liver, kidney and brain did not reveal any pathological differences between heterozygotes and homozygotes when compared to wild-type controls (data not shown). However, the testes of homozygous Cx43^26/26 ^mice weighed only about one fourth of those from wild-type males (100%) and Cx43^26/26 ^male as well as female mice were infertile as demonstrated by mating them with wild-type animals. Histological investigations revealed that, compared to the wild-type controls (Figure 5A), the differentiated stages of spermatogenesis were absent in knock-in mice, although tubuli seminiferi were developed (Figure 5D). Spermatogonia were still present, and sporadically primary spermatocytes were observed but neither spermatozoa nor other stages of spermatogenesis were found (compare Figure 5B with 5E). As a consequence, no sperms were detected in the lumen of the ductuli epididymidis (Compare Figure 5C, with Figure 5F). Since it was shown that the Leydig cells are connected by Cx43 [19], and impaired spermatogenesis could be due to a decreased number of testosterone producing cells and the presence of the androgen receptor. Immunolabeling of the androgen receptor revealed that specifically stained Leydig cells were present in a similar amount as in the controls (compare Figure 5A with 5B,E). Most of the seminiferous tubules were only lined by Sertoli cells (Figure 5D,E) but like in the wild-type controls (Figure 5B), Sertoli cells of the Cx43^43/26 ^testes (Figure 5E) expressed the androgen receptor. Finally, intratesticular testosterone levels were normal in homozygous Cx43^26/26 ^mice as compared to controls (data not shown).

**Figure 6:**
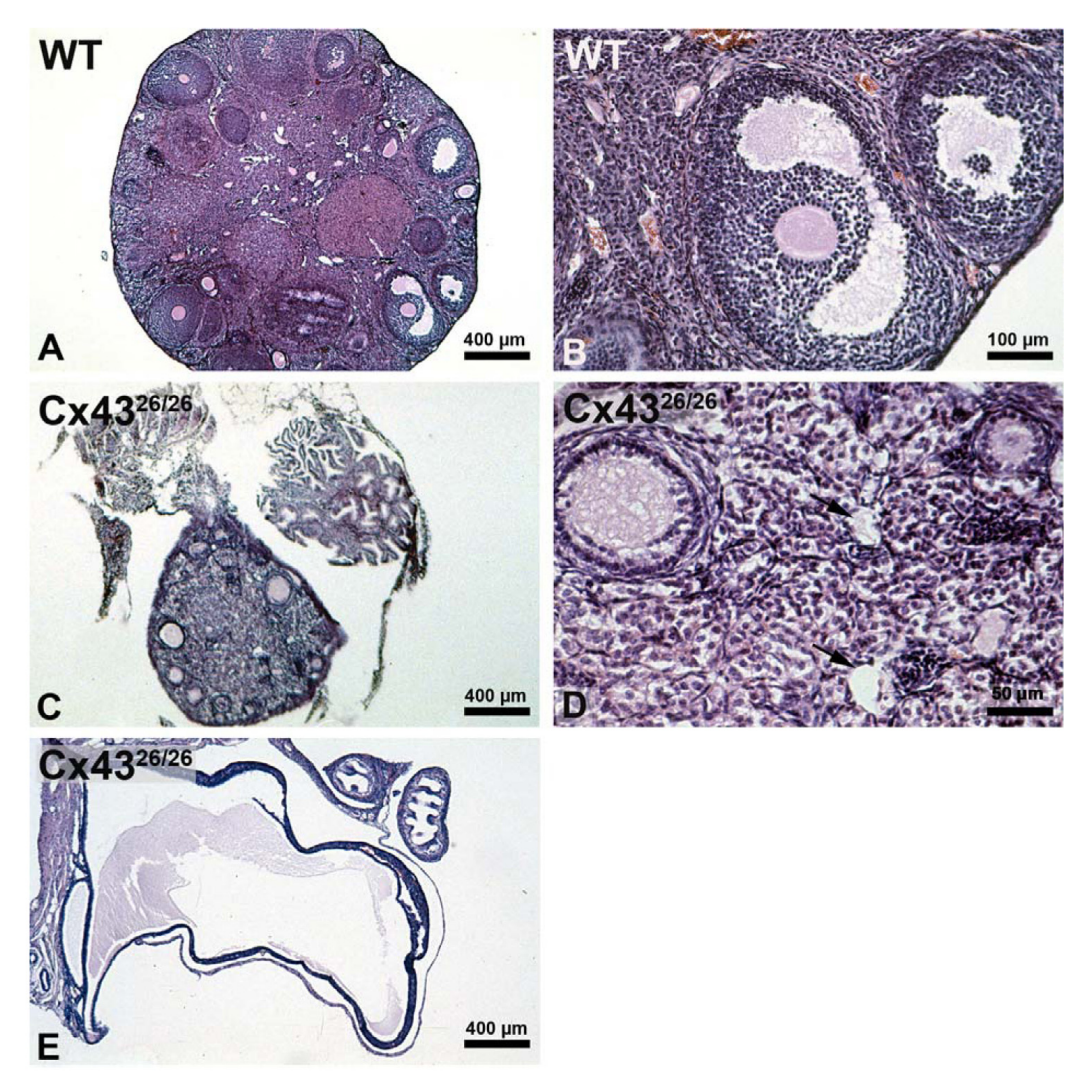
In contrast to wild-type ovaries (A, B) homozygous Cx43^26/26 ^knock-in mice reveal only few follicles and no corpora lutea (C). Maturation of follicles appears to be arrested at the early secondary stage (D). The morphology of most follicles was impaired and showed a cyst like structure (D, arrows). Some ovaries consisted of one big cyst (E).

Compared to the ovaries of wild-type animals (Figure 6A,B) the ovaries of homozygous Cx43^26/26 ^mice were obviously smaller and exhibited only few follicles (Figure 6C). The full range of follicle stages was missing and corpora lutea were absent. The maturation of the few follicles appeared to be arrested at the early secondary stage (compare Figure 6A and 6B with Figure 6C and 6E). In some cases the morphology of the follicles was impaired, i.e. in most follicles the oocytes were missing, and instead of an oocyte and follicle cells, a cyst like structure was observed (Figure 6C). In an extreme case, the complete ovary was displaced by one big cyst (Figure 6E).

**Figure 7:**
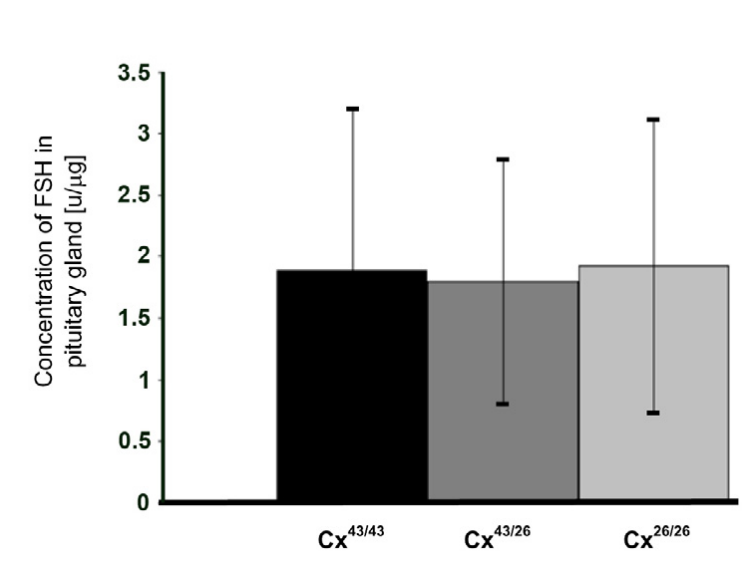
Measurements of FSH in pituitary gland: No changes in the concentration of FHS in the pituitary gland of the different genotypes were found. The weights of the pituitary glands were not different from each other. The following numbers of mice were tested: Cx43^43/43 ^(n = 10), Cx43^43/26 ^(n = 12) and Cx43^26/26 ^(n = 14).

Since this effect could be ascribed to an impaired release of follicle stimulating hormone (FSH) of the anterior pituitary gland, we investigated the FSH levels in homogenates of the pituitary gland. FSH hormone levels showed high interindividual variations but did not significantly differ between pituitary glands of Cx43^26/26 ^mice when compared with heterozygous or wild-type animals (Figure 7).

### Cardiac effects of replacing Cx43 with Cx26

Cx43 is the most abundantly expressed connexin isoform in atrial and ventricular working myocardium of mice. Its loss in Cx43-deficient mice resulted in dysmorphogenesis and obstruction of the right ventricular outflow tract, leading to early postnatal death [6]. The hearts of adult heterozygous and homozygous Cx43^26/26 ^mice showed no structural defects like those in Cx43 deficient mice except of the small size of homozygous Cx43^26/26 ^hearts, in comparison to control animals (see Additional file 1). Cx43 deficient neonates revealed significantly reduced heart rates as compared to littermates (Cx43^26/26^: 300 ± 70 bpm, Cx43^43/26^: 329 ± 67 bpm, Cx43^43/43^: 346 ± 80 bpm, p < 0.05). ECG parameters revealed no significant differences. P durations were 12 ± 3 ms (Cx43^26/26^), 13 ± 3 ms (Cx43^43/26^) and 10 ± 2 ms (Cx43^43/43^), respectively. PR intervals were 54 ± 14 ms (Cx43^26/26^), 60 ± 22 ms (Cx43^43/26^) and 52 ± 19 ms (Cx43^43/43^), respectively. QRS durations were 17 ± 3 ms (Cx43^26/26^), 15 ± 4 ms (Cx43^43/26^) and 14 ± 3 ms (Cx43^43/43^). QT and QT_c _intervals were 36 ± 10 ms and 24 ± 5 ms (Cx43^26/26^), 37 ± 13 ms and 25 ± 6 ms (Cx43^43/26^), 33 ± 10 ms and 22 ± 5 ms (Cx43^43/43^), respectively. No significant differences were obtained for left-ventricular mass (18 ± 6 mg (Cx43^26/26^), 20 ± 6 mg (Cx43^43/26^), 21 ± 6 mg (Cx43^43/43^), respectively) and ejection fraction (80 ± 9 % (Cx43^26/26^), 82 ± 9 % (Cx43^43/26^), 85 ± 8 % (Cx43^43/43^), respectively) in mice at P10. Left-ventricular mass normalized to body weight was not significantly different in neonatal mice.

Adult Cx43^26/26 ^mice had a significantly lower body (20 ± 2 g) and heart weight (95 ± 24 mg) as compared to heterozygous (Cx43^43/26^: 31 ± 3 g and 168 ± 25 mg, respectively) and control mice (Cx43^43/43^: 31 ± 3 g, 121 ± 26 mg, respectively). Heart rate and left-ventricular ejection fraction did not differ significantly between Cx43^26/26^- (456 ± 42 bpm and 85 ± 7 %), Cx43^43/26^- (524 ± 50 bpm and 84 ± 3 %) and Cx43^43/43^-mice (480 ± 80 bpm and 82 ± 9 %). Compared to their WT-littermates, Cx43^26/26 ^mice exhibited prolonged QRS- (9.1 ± 0.7 ms versus 10.9 ± 0.6 ms; P = 0.0075) and interestingly shorter rate-corrected (Mitchell) QTc-intervals (16.0 ± 2.6 ms versus 12.2 ± 1.1 ms; P = 0.0284) in the surface ECG. Infra-Hisian conduction was impaired (HV-interval: 12.4 ± 2.4 ms versus 9.5 ± 0.7 ms; P = 0.0265 ms, further supporting the presence of relevant conduction disturbances of the ventricular specific conduction system. No further significant differences were found regarding surface-ECG and invasive intracardiac electrophysiological parameters. In 4 Cx43^26/26 ^mice we found peripheral low voltage ECGs, and 2 of them died due to hemodynamical detrimental ventricular fibrillation induced by ventricular stimulation protocols.

## Discussion

This study demonstrates that Cx26 can at least in part functionally replace Cx43 in the mouse genome because heterozygous Cx43KI26 mice were viable and gave birth to viable, homozygous mutant offspring when crossed with each other.

However, only 17% of homozygous Cx43^26/26 ^mutants instead of 25%, according to Mendelian inheritance, were born and only 6% survived to day 21 post partum or longer. After interbreeding of heterozygous Cx43^43/26 ^mice, about 50% of the progeny died during the first three weeks. This phenomenon was not observed when wild-type mothers were crossed with Cx43^43/26 ^males under the same conditions, suggesting a milk delivery problem of the mothers. Histological analysis of heterozygous Cx43^43/26 ^mothers confirmed the secretory transformation of alveolar epithelium indicative of normal milk production, but revealed a less arborated ductal system on which secretory terminal end buds could develop. This could be the cause for reduced milk production in heterozygous Cx43^43/26 ^mothers. The endocrine control of lactation is one of the most complex physiological mechanisms. Estrogen, progesterone, placental lactogen, prolactin, and oxytocin act directly on the mammary gland to regulate developmental changes or coordinate milk delivery to the offspring. Prolactin is the key hormone of lactation and seems to be the single most important galactopoietic hormone, whereas oxytocin is the most powerful galactokinetic hormone [20]. Moreover, the pituitary growth hormone (GH) binds to growth hormone receptors in the stromal compartment of the mammary gland and stimulates Insulin-like growth factor-I (IGF-I) mRNA expression. GH-induced IGF-I is of major importance in ductal morphogenesis, and seems to be necessary for later stages of mammary development [21]. The sparse branching of the milk ductuli could be due to missing prolactin or growth hormone from the anterior pituitary gland which was shown to express Cx43 [22]. Anterior pituitary Cx43 levels were at maximal level during periods of high prolactin secretion. However, reduced estrogen or progesterone levels in heterozygous females cannot be excluded as a reason for impaired ductal morphogenesis. An impaired cooperation between myoepihelial and glandular cells, as it has been proposed for the heterozygous Cx43^43/32 ^mice which revealed only milk ejection problems but no dysmorphogenesis of the mammary glands [9], could not be confirmed with Cx43^43/26 ^mice. Thus, the replacement of one allele by Cx26 in the Cx43 locus seems to play a different role for mammary development during lactation as it has been shown for Cx43^43/32 ^mice. Further analyses will be needed to pinpoint the defect in lactating mammary glands of Cx43^26/26 ^mice.

Decreased levels of growth hormone could also explain our observation that homozygous animals showed a reduced body size without pathological phenotype, even if nourished by foster mothers. The gonads of both male and female homozygous Cx43^26/26 ^mice, however, were hypotrophic compared to other organs. Neither in testes nor in ovaries, complete gametogenesis was found. Due to the smaller gonads and impaired gametogenesis we investigated whether these animals suffer from a defective release of the follicle stimulating hormone from the pituitary gland which regulates both follicle maturation and spermatogenesis. However, our measurements of FSH did not confirm this hypothesis. The presence of the androgen receptor in both Leydig and Sertoli cells is not consistent with impaired spermatogenesis leading to a kind of "Sertoli-cell-only" syndrome. Our results indicate that Cx26, similar to Cx32 [9], cannot functionally replace Cx43 which is abundantly expressed between Sertoli cells. Since spermatogonia are present and even start to enter spermatogenesis, this indicates a direct nourishing role for Cx43 in Sertoli cells in this differentiation process. The finding that functional expression of Cx43 gap junction channels is necessary for spermatogenesis confirms the investigations of Roscoe et al. [8]. These authors recognized a "Sertoli-cell-only" syndrome in testes which lacked Cx43 and were cultivated under the kidney capsule, although the production of testosterone was similar to wild-type testes.

Replacing Cx43 with Cx26 in ovarian follicles led to an arrested growth of less follicles up to the secondary stage and a cystic transformation. Like in the Cx43^26/26 ^mice, the ovaries of Cx43 deficient mice, when grafted under the kidney capsule [23] did not develop beyond the primary stage and revealed less follicles. Thus, Cx26 channels between granulosa cells were not able to maintain folliculogenesis, and its expression led to a similar defect as shown in Cx43 null mouse mutants. Again, this is different from the observation in Cx43^32/32 ^mice which showed normal ovarian histology and could become pregnant [9]. Thus, in contrast to Cx26 which cannot replace the function of Cx43 in folliculogenesis and spermatogenesis, the Cx32 protein is able to take over the function of Cx43 in granulosa cells for oocyte maturation.

Although replacement of Cx43 by Cx26 leads to gonadal dysfunction in male and female, Cx26 can apparently partially replace Cx43 in working myocardium, at least in short-term function during several postnatal weeks, similar as previously described for Cx43KI32 and Cx43KI40 mice [9]. Cx43^26/26 ^mice seemed to develop similar electrophysiological abnormalities, although slower than Cx43^-/- ^mice. We noticed a prolonged QRS complex as a marker for possible bundle branch blocks or longer depolarization phase and a tendency towards ventricular arrhythmia as described in Cx43 deficient mice [24]. Interestingly, the QTc interval was shorter in the present mouse model. Thus, the repolarization phase did not seem to be affected in a similar way. Repolarization characteristics should therefore be further investigated using optical or electrical mapping systems. Despite this, in vivo electrophysiological investigation showed that Cx26 cannot completely compensate for Cx43 missing in conductive and working myocardium. The strongly decreased survival of Cx43^26/26 ^mice suggests that the long-term function of the myocardium is impaired.

## Conclusion

Finally, we found differences in the ability of Cx32 and Cx26 to functionally replace Cx43 in the lactating mammary gland. Both heterozygous knock-in mice demonstrated impairment of mammary function but in a different way. Replacement of Cx43 by Cx32 disturbed milk ejection, whereas replacement of Cx43 by Cx26 led to a dysmorphogenesis of gland arboration. These results extend previous observations with Cx43 knock-in mice [9] that the different connexin isoforms fulfill individual roles in certain cell types but can at least partially replace each other in other cell types.

## Methods

### Generation of mice

A fragment of genomic 129/Sv mouse DNA spanning about 12 kb of the Cx43 locus was isolated as described previously [9] from a recombinant lambda phage library (Stratagene La Jolla, USA). The Cx43KI26 construct was embedded in a pBluescriptII SK+ vector backbone (Stratagene). A 5 kb *Sac*I-*Nco*I fragment and a 1.6 kb *Cla*I-*Ava*I fragment were used as 5' and 3' homologous regions, respectively.

The coding region of Cx26 was cloned by introduction of a NcoI restriction site into the ATG translational start codon of Cx43. For this purpose, 1.1 kb of the 5' homologous region and the coding region of Cx26 were amplified by PCR using modified primers. The coding region of Cx26 was cloned behind 1.95 kb of the phosphoglycerate kinase (PGK) promoter-driven neomycin selection cassette flanked by two frt sites. The final targeting vector pCx43KI26 was analyzed by restriction mapping and partial sequencing. The function of frt sites was verified by transformation of Flp recombinase expressing E. *coli *bacteria [25].

HM-1 embryonic stem cells were transfected with 250 μg DNA of the targeting vector pCx43KI26, linearized upstream of the 5' homologous region by NotI and selected with 350 μg G418 (Sigma, St. Louis, MO) per ml of medium as described previously [26,27]. Resistant clones were analyzed for homologous recombination by PCR and Southern blot hybridization. Correctly recombined ES clones were injected into C57BL/6 blastocysts to generate chimeras as described by Nagy et al. [28]. In order to obtain germ line transmission of the mutated allele, the chimeras were crossed to C57BL/6 mice. Male heterozygous offspring, designated as Cx43KI26neo, were further crossed to female Flp mice [29] to generate the final genotype Cx43KI26. All analyses were carried out on mixed 129/Ola/C57BL/6 genetic background using littermates as controls.

Mice were kept under standard housing conditions with fixed 12 hours/12 hours light/dark circle and food as well as water ad libitum. All experiments were carried out in accordance with the German law for animal welfare and with permission of local state authorities.

### Genotyping of ES cells and mouse tissues

G418 resistant ES clones were prepared and processed for PCR analysis. Recombination at the 3' homologous site was examined using a neomycin specific upstream primer (KI26 neo2; 5'-GAG ACT AGT GAG ACG TGC TAC TTC-3') and a 3' downstream primer external to the targeting vector (KI26 extern; 5'-CAT ACC ATT GCA CAG AAG ATA CCG G-3'). A 2.1 kb amplicon was indicative of homologous recombination.

For genomic Southern blot hybridization, DNA from PCR-positive clones was digested by *Pst*I for the external probe and by *Hin*dIII for the internal probe. A 550 bp AvaI/AvaI fragment outside the 3' homologous region of Cx43 served as external probe. In addition, a Cx26 internal probe (775 bp) was used. After *Pst*I digestion and hybridization to the Cx43 external probe, the Cx43 wild-type allele yielded a 8 kb fragment compared to a 3.5 kb fragment of the mutated allele. The Cx26 internal probe detected a 1.3 kb fragment of the wild-type allele and a 2.0 kb fragment of the recombined allele after *Hin*dIII digestion.

For routine genotypic analysis, genomic DNAs from tail tips were used for PCR including three primers: Cx43-3'-HO2 (coding region of Cx43; 5'-CGC ATT TAC AAC AAG CAA GCC AGC-3'), Cx43-3'-RO4 (3' untranslated region of Cx43; 5'-CGC CTC ATT ACT GAG GTT GTT GAG-3') and Ki26neo5 (neomycin selection cassette; 5'-CGC AGC GCA TCG CCT TCT TCG CC-3') that generated a 381 bp amplicon of wild-type and a 529 bp amplicon of the knock-in allele.

After action of the Flp recombinase, the deletion of neomycin selection cassette was analyzed by using the primers Cx43-3'-HO2 (5'-CGC ATT TAC AAC AAG CAA GCC AGC-3'), Cx43-3'-RO4 (5'-CGC CTC ATT ACT GAG GTT GTT GAG-3') and Ki26Cx26 (5'-GGT GGA CCT ACA CCA CCA GCA TC-3'), which generated a 381 bp amplicon of wild-type and a 529 bp amplicon of the knock-in allele.

### Southern blot analysis

For Southern blot hybridization of wild-type (i.e. Cx43^43/43^), heterozygous (Cx43^43/26^) and homozygous (Cx43^26/26^) knock-in mice, genomic DNA was prepared from adult livers and digested with *Pst*I for analysis with the Cx43 external probe, as well as with *Hin*dIII for the Cx26 internal probe. After digestion, genomic DNA was fractionated on 0.6 % agarose gels and transferred onto nylon membrane (Hybond™-N+, Amersham Biosciences UK). The Cx43 external probe (487 base pairs outside of the 3' homologous region) and Cx26 internal probe (775 base pairs of Cx26 coding region) were obtained from the corresponding vector by digestion with appropriate restrictases. The probes were radioactively labelled using the Multiprime labelling system (Amersham-Bucher GmbH, Braunschweig, Germany) according to instructions of the manufacturer. Hybridization, washing and Southern blot analyses were carried out as recommended by the supplier (QuikHyb^(R) ^Hybridization Solution; Stratagene, Stadt, CA).

### Western blot analysis

Mouse hearts and brains were dissected, immediately frozen on dry ice and stored at -70°C until further use. Homogenized tissue was taken up in protein lysis buffer (60 mM Tris HCl, pH 7.4 and 3 % sodium dodecyl sulphate), supplemented with protease inhibitor "Complete" (Roche, Mannheim, Germany) and sonicated three times for 10 seconds on ice. Protein concentration was determined using the bicinchoninic acid protein assay (Sigma, Taufkirchen, Germany). Fifty μg protein were separated on 12.5 % SDS-polyacrylamide gels and electroblotted on nitrocellulose membranes (Hybond ECL, Biosciences, Bucks, UK) for 45 minutes at 100 V. Membranes were blocked for 1 hour with buffer (20 mM Tris-HCl, pH 7.4, 150 mM NaCl, 0.1 % Tween 20) and 5 % milk powder (w/v) before incubation with anti-Cx43. Afterwards the membranes were incubated with rabbit anti-Cx43 (1:2000, prepared against a peptide containing the last 14 C-terminal amino acid residues, by Christian Schlieker in our laboratory) and mouse anti-Cx26 (1:1000, Zymed) overnight at 4°C.

### RT-PCR

Mice of defined genotype, verified by PCR analysis of tailtip DNA, were killed by cervical dislocation. Brain, heart, liver and testes were homogenized in 2.5 μl TRIzol (Invitrogen, Carlsbad, CA). The total RNA was isolated according to the protocol of Invitrogen. Two microgram of total RNA were used for RT-PCR. For this purpose Oligo-dT primers and the AMV reverse transcriptase (Gibco-BRL) were used as recommended by the supplier. With 100 nanogram of cDNA, polymerase chain reactions with the following intron-spanning primers were carried out: Cx26 USP 5'-CGGAAGTTCATGAAGGGAGAGAT-3'; Cx26 DSP 5'-GGTCTTTTGGACTTTCCTGAGCA-3'; Cx43 USP 5'-TACCACGCCACCGGCCCA-3'; Cx43 DSP 5'-GGCATTTTGGCTGCTGTCAGGGAD-3'; β-Actin USP 5'-CGTGGGCCGCCCTAGCCACCAG-3'; β-Actin DSP 5'-TTGGCCTTAGGGTTCAGG-GGGG-3'. PCR was carried out using the following program: 94°C for 3 min., 35 cycles with 94°C for 1 min, 55°C for 1 min, 72°C for 2 min., and finally 72°C for 10 min. The probes were franctionated on a 2% agarose gel and the bands were visualized by ethidium bromide staining. The stained gels were scanned using the ImageMaster Program (Amersham Biosciences, Freiburg, Germany).

### Histological Analysis

Tissue samples from adult mice were fixed in PBS-buffered 4% (w/v) formaldehyde for a maximum of 24 h at 4°C. The gonads (ovary and testis) were fixed in Bouin solution (75 ml picrinic acid, 5 ml acidic acid, and 25 ml of 40% formaldehyde) and processed routinely for paraffin sections.

### Immunohistochemistry

Sections (7 μm) were prepared from Bouin's fixed and paraffin-embedded testis and rehydrated. An antigen retrieval step was performed by microwaving the sections in 0.05 M glycine buffer for 10 min (1200 W). Unspecific binding of the first antibody was blocked by 30 min incubation step in antibody diluent (DakoCytomation Carpinteria, CA, USA, S3022). Rabbit polyclonal antibodies raised against a N-terminal peptide of the androgen receptor of human origin recognizing also mouse and rat androgen receptor (Santa Cruz Biotechnology, Santa Cruz, CA, USA) were used at 1:800 dilutions in antibody diluent. DakoCytomation Universal LSAB Plus-kit (K0678) including biotinylated second antibody polymer and alkaline phosphatase conjugated streptavidin were employed for detection of bound primary antibodies according to the manufacturer's instructions. New Fuchsin chromogen was used as substrate for alkaline phosphatase. Mayer's hematoxylin was used as conterstain. Endogenous alkaline phosphatase was inhibited by addition of Levamisole to the substrate. Control stains were carried out omitting the primary antibodies.

### FSH determination

For evaluation of FSH tissue levels, the pituitary glands of adult Cx43^43/43 ^(n = 10), Cx43^43/26 ^(n = 12) and Cx43^26/26 ^(n = 14) female mice were removed and stored at -70°C. Pituitaries were sonicated to homogeneity in 1 ml of ice-cold 0.01 M phosphate-buffered saline containing 10 μl protease inhibitor cocktail (Sigma; Deisenhofen, Germany). Pituitary FSH was determined as described previously (Simoni et al. 1992), using reagents for rat FSH provided by NIDDK (Bethesda, MD). The assay is based on the FSH-RP-2 standard preparation, anti-rFSH-S-11 antiserum and FSH-I-6 for iodination. All samples were measured in one assay, with the sensitivity of 1.6 ng/ml and an intra-assay coefficient of variation of 5.8%.

### ECG recordings and echocardiography

ECG measurements were carried out on 10 days old postnatal mice of both genders (n = 52) and 11 adult mice. Mice were fixed on a heating pad warmed to maintain body temperature. A surface 6-lead ECG was acquired digitally by means of a multichannel amplifier (PowerLab™ System, ADInstruments, Milford, MA, USA) [17]. ECG channels were amplified, filtered between 10 and 100 Hz and sampled at a rate of 1 kHz per channel. Heart rate, P-wave duration, PR-interval, QRS-duration, QRS-amplitude and QT-interval were determined off-line as published recently [30]. The QT-interval was rate-corrected (QTc) according to Mitchell et al. [31]. High resolutional mouse echocardiography was performed in adult mice using a commercially available ultrasound system (HDI 5000, Philips-Ultrasound, Bothell, WA) equipped with a 15 MHz linear array transducer. Volumetric analysis of left ventricular (LV) structures was performed to assess mass and function [32,33]. Structures which are known to be prone to congenital defects in Cx43-deficient mice, in particular, the right-ventricular outflow tract, were investigated carefully by B-mode and pw-Doppler.

Echocardiographic parameters were compared between the three genotypes by means of one-way ANOVA along with post-hoc Tuckey-Kramer Multiple Comparisons Test. P-values < 0.05 for ANOVA were considered as significant. Results of post-hoc analyses of heterozygous (HT) or wild type (WT) versus homozygous (HO) are symbolized as follows: * = P < 0.05, ** = P < 0.01, *** = P < 0.001. For comparison of two groups, 2-sided Student's t-test was performed. P-values < 0.05 were considered as significant.

### Electrophysiological investigations

Seven Cx43^26/26 ^mice and 5 WT-littermates were electrophysiologically characterized in vivo by invasive single-catheter investigation as previously described [34]. In short, preparation and catheterization (octapolar electrophysiologic catheter, Ciber Mouse, NuMed, NY, USA) of the jugular vein and electrophysiologic investigation were performed under inhalative anaesthesia (1,5 vol% isoflurane in 70 % N_2_O and 30 % O_2_) at a constant body temperature of 37°C. The electrophysiological investigation included intracardiac atrial and ventricular stimulation. Baseline ECG (R-R interval, P-wave duration, PQ interval, QRS duration, QT interval) and electrophysiologic parameters (sinus nodal refractory time, Wenckebach-periodicity, 2:1 atriventricular conduction, AV-nodal refractory period, VA-conduction properties and refractory periods) were evaluated. Inducibility and incidence of atrial and ventricular tachyarrhythmias were tested using programmed (up to 3 extra-beats) and burst stimulation protocols as previously described (Schrickel et al., 2002) The pacing threshold current was 1.01 ± 0.56 mA at 1 ms stimulus duration. Twice pacing threshold rectangular stimulus pulses were administered by a modified programmable stimulator with S1S1 cycle lengths (CL) down to 10 ms (Model 5328; Medtronic, Minneapolis, MN, USA). Atrial fibrillation lasting >1 sec. was evaluated, ventricular tachycardia was defined as >4 ectopic ventricular beats. After amplification and filtering, data were sampled with a rate of 4 kHz (Bard stamp amplifier; Bard LabSystem, C.R. Bard Inc., New Jersey, USA) and stored on optical discs. Statistical analysis was performed using a two-sided t-test and a P < 0.05 was regarded as statistically significant.

## Authors' contributions

E.W. and K.W. designed research. E.W. evaluated the phenotype of reproductive organs. N.P. generated mice, performed genotyping, analyzed data. K.M., S.U., J.F. helped to generate mice. R.B., R.G. performed IHC. A.G., J. W.S., T.L., K.T. performed ECG recordings and echocardiography. J.S.K. evaluated the heart phenotype. M.S. performed FSH determination. E.W., K.W. wrote the paper. All authors read and approved the final manuscript.

## Supplementary Material

Additional file 1 Heart morphology. Histology of wild-type (Cx43^43/43^), heterozygous (Cx43^43/26^) and homozygous Cx43^26/26^) hearts. Besides the difference in size, no histological differences were found. ra: right atrium, la: left atrium, rv: right ventricle, lv: left ventricle. Click here for file
