# Supplementary figures and images for: Replacement of connexin43 by connexin26 in transgenic mice leads to dysfunctional reproductive organs and slowed ventricular conduction in the heart
Source: BMC Dev Biol. 2007 Apr 4;7:26. doi: 10.1186/1471-213X-7-26 (PMC1852306; doi:10.1186/1471-213X-7-26)

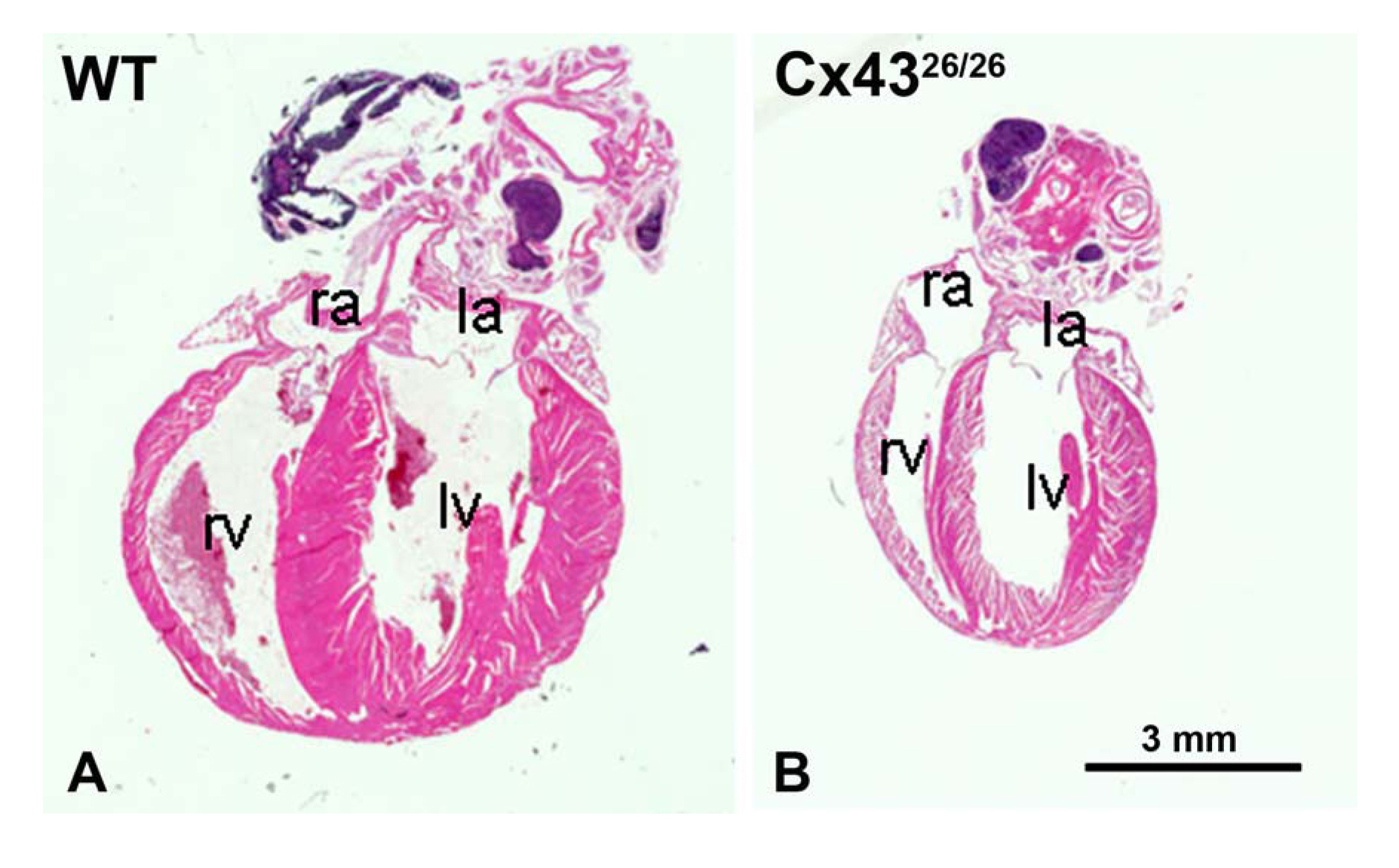

Supplement: Additional file 1 — Heart morphology. Histology of wild-type (Cx4343/43), heterozygous (Cx4343/26) and homozygous Cx4326/26) hearts. Besides the difference in size, no histological differences were found. ra: right atrium, la: left atrium, rv: right ventricle, lv: left ventricle. [file 1471-213X-7-26-S1.jpeg]
